# Therapeutic Effects of Novel Sphingosine-1-Phosphate Receptor Agonist W-061 in Murine DSS Colitis

**DOI:** 10.1371/journal.pone.0023933

**Published:** 2011-09-08

**Authors:** Yasuaki Sanada, Tsunekazu Mizushima, Yasuyuki Kai, Junichi Nishimura, Hiroshi Hagiya, Haruto Kurata, Hirotaka Mizuno, Etsuko Uejima, Toshinori Ito

**Affiliations:** 1 Departments of Complementary and Alternative Medicine, Graduate School of Medicine, Osaka University, Osaka, Japan; 2 Department of Hospital Pharmacy Education, Graduate School of Pharmaceutical Science, Osaka University, Osaka, Japan; 3 Department of Surgery, Gastroenterological Surgery, Graduate School of Medicine, Osaka University, Osaka, Japan; 4 Minase Research Institute, Ono Pharmaceutical Co., Ltd, Osaka, Japan; 5 Tsukuba Research Institute, Ono Pharmaceutical Co., Ltd, Ibaraki, Japan; Ulm University, Germany

## Abstract

Although IL-17 is a pro-inflammatory cytokine reportedly involved in various autoimmune inflammatory disorders, its role remains unclear in murine models of colitis. Acute colitis was induced by 2.5% dextran sodium sulfate (DSS) treatment for 5 days. A novel sphingosine-1-phosphate receptor agonist W-061, a prototype of ONO-4641, was orally administered daily, and histopathological analysis was performed on the colon. The number of lymphocytes and their cytokine production were also evaluated in spleen, mesenteric lymph node, Peyer's patch and lamina propria of the colon. Daily administration of W-061 resulted in improvement of DSS-induced colitis, and significantly reduced the number of CD4+ T cells in the colonic lamina propria. Numbers of both Th17 and Th1 cells were reduced by W-061 treatment. W-061, however, had no influence on the number of Treg cells in lamina propria. Thus, Th17 and Th1 cells in lamina propria were thought to be the key subsets in the pathogenesis of DSS-induced colitis. In conclusion, W-061 may be a novel therapeutic strategy to ameliorate acute aggravation of inflammatory bowel diseases.

## Introduction

Inflammatory bowel diseases (IBDs), such as Crohn's disease (CD) and ulcerative colitis (UC), are chronic relapsing disorders of the gastrointestinal tract [1]. In humans, CD is generally characterized by elevated production of helper T (Th) 1 cytokines, such as interferon (IFN)-γ [2], while UC is characterized by the enhanced expression of Th2 cytokines, such as interleukin (IL)-4 and IL-13 [3]. Recent clinical data have indicated that treatment with infliximab, a chimeric anti-tumor necrosis factor (TNF)-α antibody, was further effective as compared to azathioprine therapy in patients with CD [4]. Despite its efficacy, infliximab therapy for patients with rheumatoid arthritis (RA) was associated with the risk of malignancies and serious infections, such as tuberculosis [5]. Therefore, novel therapeutic strategies with different mechanisms are required.

In recent years, Th17, a third subset of inflammatory helper T cells that produce IL-17A, IL-17F, IL-22 and IL-23, was discovered [6]. Th17 has been reported to be associated with aggravation of various autoimmune inflammatory diseases, such as rheumatoid arthritis and multiple sclerosis [7]-[9]. Although IL-17 expression in the mucosa and its serum levels were increased in active IBD patients [10], treatment strategies for the regulation of Th17 functions has not yet been established.

It has been reported that serum IL-17 concentration is elevated in the acute phase in a dextran sodium sulfate (DSS)-induced colitis model [11], however, the role of IL-17 remains unclear in animal models of colitis. For example, controversial data have been obtained in the DSS-induced colitis model [12], [13] and CD4^+^ CD45RB^high^ adoptive cell transfer model [14], [15].

In contrast, regulatory T (Treg) cells are a distinct subset in suppressing excessive immune responses [16]. This subset is characterized by the presence of a surface marker, CD25, and a transcription factor, Foxp3. In a CD4^+^ CD45RB^high^ cell-transfer colitis model, the development of colitis was suppressed by concomitant transfer of CD4^+^ CD25^+^ T cells [17].

It was recently reported that sphingosine-1-phosphate (S1P) receptor agonists, such as FTY720 (fingolimod), exhibited immunodulatory function through induction of CD4^+^ T cell migration into secondary lymphoid tissues and sequestration of these cells [18] and that FTY720, which has a particular binding affinity for S1P_1_, was clinically effective in the treatment of multiple sclerosis [19]. FTY720 was also reported to be effective for the treatment of colitis in IL-10-deficient mice [20], DSS-induced colitis and CD4^+^CD62L^+^ cell-transfer model [21]. Another S1P receptor agonist, KRP-203, was also useful for colitis in IL-10 deficient mice [22]. However, the effects of these agents on Th17 and Treg in colitis mice have not been examined and reported to date.

W-061, a prototype of ONO-4641 [23], has recently been developed to target S1P receptors. In the present study, we evaluated the activity of W-061 on S1P receptors and the effects of this agent on specific T cells, such as Th17 and Treg, in a mouse model of DSS-induced colitis.

## Results

### W-061 has specific agonistic activity on hS1P_1_

Compared to FTY720 phosphate, binding affinity of S1P receptors and agonistic activity of W-061 was evaluated in CHO-K1 cells. W-061 bound to human S1P receptors except for hS1P_2_ (Table 1). Its binding affinity of S1P receptors was lower than that of FTY720 phosphate, however, W-061 had higher agonistic activity on S1P_1_ compared to S1P_3_ (Table 2), while FTY720 phosphate had agonistic activity on S1P_3_ as well as S1P_1_.

**Table 1:** Binding affinity of W-061 on S1P receptors.

	hS1P_1_	hS1P_2_	hS1P_3_	hS1P_4_	hS1P_5_
S1P	0.131	0.439	0.0782	7.60	0.372
FTY720-P	0.160	4090	3.74	2.16	1.09
W-061	4.11	>43800	1710	65.4	10.1

Data were shown as Ki value. FTY720-P; FTY720-phosphate.

**Table 2:** Agonistic activity of W-061 on S1P_1_ and S1P_3_.

	hS1P_1_	hS1P_3_
S1P	4.7	30
FTY720-P	0.23	20
W-061	10	>30000

### Body weight and colon length were improved by treatment of W-061

**Figure 1:**
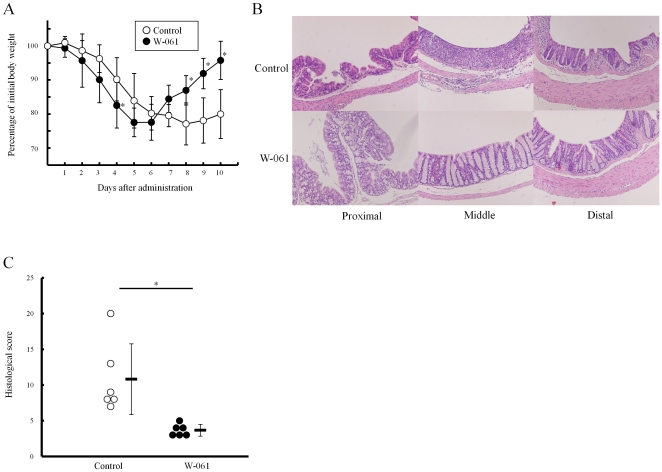
DSS-induced colitis was ameliorated by W-061 treatment. (A) Change in body weight of W-061-treated and control mice with the DSS-induced colitis (n = 6 mice for each group, *p<0.05). (B) Pathological evaluation of the DSS-induced colitis on day 10. (C) Histological score of the DSS-induced colitis on day 10. Data represent absolute values (means ± SD, n = 6 mice for each group, *p<0.05).

No change of body weight was observed in mice treated with W-061 (3 mg/kg) alone (Figure S1). After 5-day DSS treatment, body weight in each group was reduced by approximately 20% (Figure 1A). After day 7, however, a significant body weight increase was observed in W-061-treated mice (95.8±5.6% vs. 80.0±7.2% on day 10). DSS treatment induced a reduction in colon length in each group; however, colon length in W-061 treated mice was significantly longer than in control mice on day 10 (10.8±0.6 cm vs. 9.1±1.6 cm, *p*<0.05).

### Daily W-061 administration significantly promoted recovery from acute DSS-induced colitis

Histological analysis indicated that cellular infiltration in the lamina propria, mucin depletion and thickness of the mucosa were severe in control mice when compared with W-061 treated mice (Figure 1B). Mean histologic score in mice treated with W-061 was significantly lower than in control mice (Figure 1C: 3.7±0.6 vs. 10.8±4.5).

### Daily W-061 administration induced homing of lymphocytes to secondary lymphoid tissues

**Figure 2:**
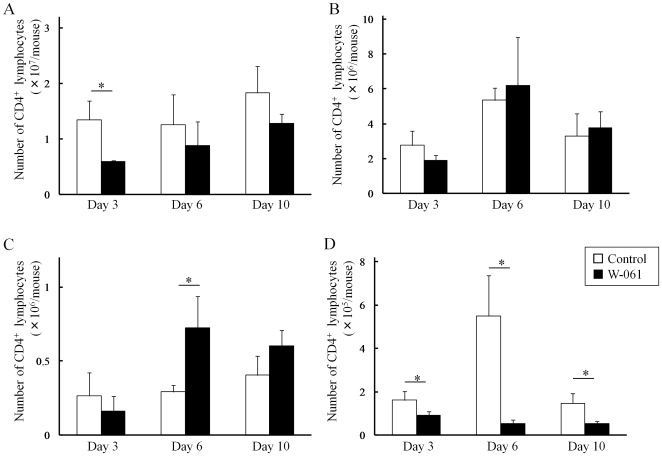
Change of the numbers of CD4^+^ T cells in (A) SP, (B) MLN, (C) PP and (D) LP. Data represent absolute values (means ± SD, n = 4 per group on days 3 and 6, n = 6 per group on day 10, * p<0.05).

The number of lymphocytes in LP was sequentially increased by DSS treatment. However, W-061 administration suppressed lymphocyte migration to SP and LP, and promoted their homing to secondary lymphoid tissues, such as MLN and PP (Figure 2A–D). Specifically, W-061 inhibited CD4^+^ T cell migration to LP on day 3, 6 and 10 (Figure 2D).

### Daily W-061 administration inhibited migration of Th17 and Th1 to lamina propria

**Figure 3:**
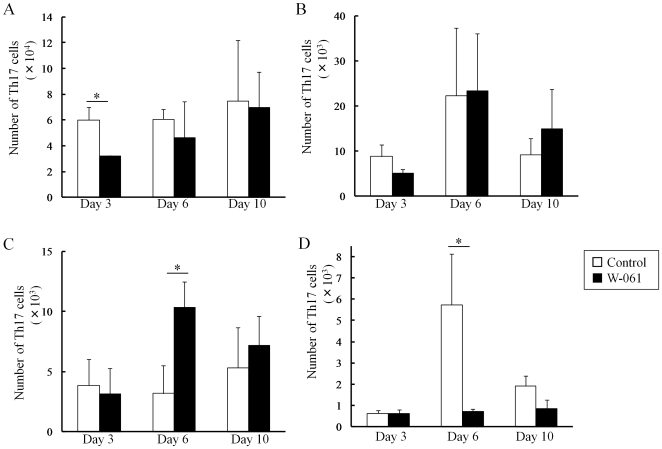
Change of the numbers of Th17 cells in (A) SP, (B) MLN, (C) PP and (D) LP. Data represent absolute values (means ± SD, n = 4 per group on days 3 and 6, n = 6 per group on day 10, * p<0.05).

**Figure 4:**
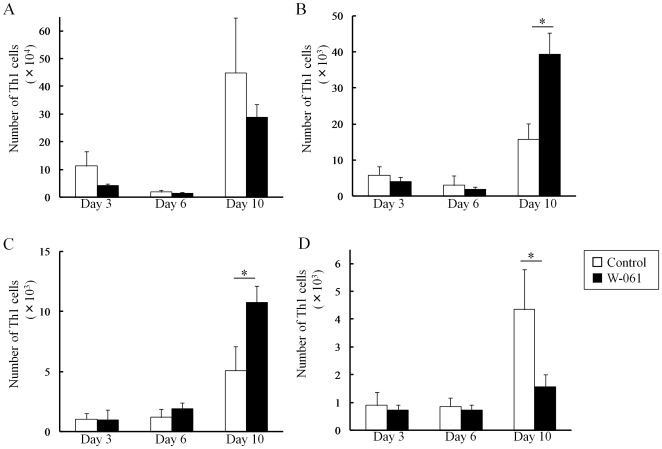
Change of the numbers of Th1 cells in (A) SP, (B) MLN, (C) PP and (D) LP. Data represent absolute values (means ± SD, n = 4 per group on days 3 and 6, n = 6 per group on day 10, * p<0.05).

**Figure 5:**
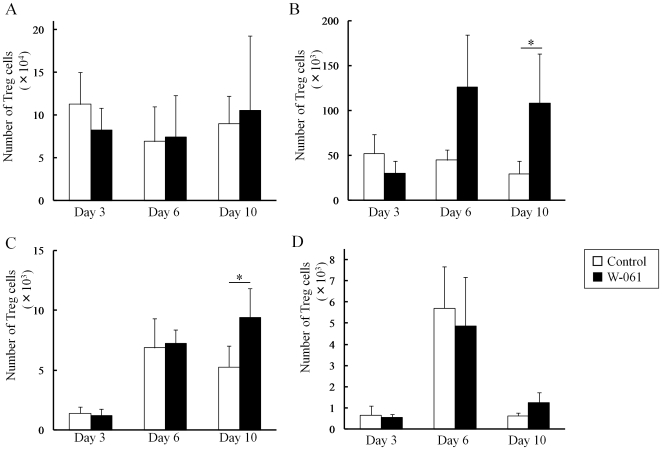
Change of the numbers of Treg cells in (A) SP, (B) MLN, (C) PP and (D) LP. Data represent absolute values (means ± SD, n = 4 per group on days 3 and 6, n = 6 per group on day 10, * p<0.05).

In the control group, an increase in the number of Th1 cells in LP was observed following the increase in the number of Th17 cells. On the other hand, W-061 treatment inhibited the increase in Th17 cells on days 6 and 10 (Figure 3D). In addition, W-061 also inhibited the increase in Th1 cells on day 10 (Figure 4D). Instead, Th17 cells increased in PP on day 6, and Th1 cells increased in MLN and PP on day 10. The number of Treg cells in both groups increased on day 6 (Figure 5D); however, there was no difference in the change in Treg cells. Changes in the number of Th1, Th17 and Treg cells were also confirmed in SP, MLN and PP (Figure 5A–C). In PP, the number of Th17 cells was significantly higher in the W-061 group on day 6, and the numbers of Th1 and Treg cells were significantly higher on day 10 (Figure 4C, 5C). Similarly, in MLN, the numbers of Th1 and Treg cells were also significantly higher in W-061 group on day 10 (Figure 4B, 5B).

## Discussion

In the clinical course of IBD, patients occasionally become immunocompromised due to treatment with immunosuppressive reagents, such as infliximab, steroids, antimetabolites and calcineurin inhibitors. It has been reported that RA patients receiving infliximab can develop serious opportunistic infections, such as tuberculosis [5]. In terms of the potent properties in anti-opportunistic infections [24] and anti-tumor effects [25], [26], we have been investigating the efficacy of S1P receptor agonists in promoting lymphocyte homing and sequestrating lymphocytes into secondary lymphoid organs. We previously reported the usefulness of FTY720 and KRP-203 in IL-10-deficient mice as a CD-like colitis model [20], [22]. In this study, a novel and potent S1P receptor agonist, W-061, which is structurally different from sphingosine, was examined. In contrast to conventional S1P receptor agonists, W-061, which is biologically active *in vivo* without undergoing phosphorylation, is more specific to lymphocytes than FTY720.

We first evaluated the membrane binding activity of W-061 to S1P receptors, which has five subtypes (S1P_1-5_) [18]. The specific roles of S1P receptor subtypes have been reported, with S1P_1_ and S1P_4_ being strongly expressed in T cells [27]. S1P_1_ is particularly associated with lymphocyte migration. In some clinical reports of FTY720, symptomatic bradycardia was noted as an adverse event [28], and this is related to agonistic activity of S1P_3_ [29]. W-061 was found to have a lower affinity for S1P_1_, but to have a much lower affinity for and S1P_3_ than FTY720 phosphate (Table 1). Thus, it could be considered that W-061 had a higher selectivity for S1P_1_ than FTY720 phosphate. In addition, W-061 had little agonistic activity on S1P_3_ (Table 2). Taken together, these properties may be helpful not only for adjusting lymphocyte trafficking with less adverse events such as symptomatic bradycardia.

The recent advances in our knowledge of the immunopathologic basis of IBD have been approaching the elucidation of the inflammatory cytokines driving the two main components of IBD, CD and UC. CD patients, but not UC patients, exhibit elevated amounts of IFN-γ in the inflamed LP, suggesting the presence of a Th1-mediated inflammatory process. Recently, a novel helper T cell, Th17, was identified, and was reported to be a proinflammatory subset in several autoimmune diseases [7]–[9]. In the case of 2,4,6-trinitrobenzenesulfonic acid (TNBS)-induced colitis, it was also shown to be driven by a cytokine including IL-12p40 and to be reversed by anti-IL-12p40 mAb [30]. Furthermore, it was found that a novel cytokine, IL-23, shared the common p40 chain with IL-12 [31]. It is generally recognized that Th17 cells are induced by IL-23 together with TGFβ, while Th1 cells are induced by IL-12 or IL-23 without TGFβ [6]. Therefore, anti-IL-12p40 mAb inhibits Th17 as well as Th1.

Although the role of Th1 is reported to be an indispensable subset [32], the role of Th17 remains controversial in DSS-induced colitis or the CD4^+^ CD45RB^high^ lymphocyte transfer model. In the case of DSS-induced colitis, inflammation is improved in IL-17-deficient mice [12], but is aggravated by neutralization of IL-17 due to anti-IL-17 mAb [13]. In the adoptive transfer model, transfer of RORγ-null T cells do not induce colitis [14]; however, lymphocytes from IL-17A-deficient mice induced severe colitis [15]. Thus, the type of colitis model or the time of inflammation may influence whether Th1 or Th17 cells contribute the pathogenesis of colitis.

In the previous reports, however, we did not evaluate the effects of S1P receptor agonists on the new T cell subsets, such as Th17 and Treg. In order to investigate the effect of W-061 on these T cells, we utilized a DSS-induced mice colitis model which does not exhibit any immune disorder, in contrast to IL-10 deficient mice. Treg cells cannot be evaluated in IL-10-deficient mice because IL-10 is necessary for the effector function of the regulatory T cell population [32]. We showed that the number of Th17 cells in LP was markedly higher after the initiation of colitis, and then decreased significantly, which appears to be due to the induction of Treg cells. After downregulation of Th17 cells, it is likely that IFN-γ-producing Th1 cells prolong the inflammation in LP.

In contrast, when W-061 was administered, the number of Th1 and Th17 cells was markedly decreased in the acute phase of inflammation. Interestingly, W-061 did not influence the migration of Treg cells. These data suggest that W-061 promotes the migration of Th17 and Th1 cells, but not Treg cells into secondary lymphoid tissues. It is generally known that S1P receptor agonists do not affect migration of memory T and B cells into secondary lymphoid tissues, resulting in the maintenance of the anti-opportunistic infections or anti-tumor effects [24]-[26]. The chemokine receptor CCR7 is reported to be a major homing receptor in T cells [33]. Although CCR7 is expressed in Treg cells, as well as other T cell subsets, its effects differ between naïve-like Treg and effector/memory-like Treg [34]. Namely, naïve like Treg can enter themelves into lymph node via CCR7 to control the priming phase of immune responses. On the other hand, CCR7 on effector/memory Treg is associated to emigrate themselves from the inflamed site. In terms of lymphocyte homing, W-061 is likely to have some selectivity. Considering that accumulation of effector/memory-like Treg at inflamed sites was induced by CCR7 deficiency [34], W-061 may affect CCR7 expression. Further study is thus needed to clarify whether migration of Treg via S1P_1_ receptor is different from that of other T cell subsets.

In summary, we herein demonstrate that a novel S1P receptor agonist W-061 is effective in the treatment of acute colitis induced by DSS. This effect is attributed to the suppression of increases in Th17 and Th1 in LP by the sequestration of these cells into the secondary lymphoid tissues. Thus, Th17 and Th1 in LP are a key subset in the pathogenesis of DSS-induced colitis. S1P receptor agonists, including W-061, may therefore represent a novel therapeutic strategy for ameliorating acute aggravation of IBDs.

## Materials and Methods

### Cell Culture

CHO-K1 cells stably expressing human S1P_1_ (hS1P_1_), human S1P_2_ (hS1P_2_), human S1P_3_ (hS1P_3_), human S1P_4_ (hS1P_4_) or human S1P_5_ (hS1P_5_) were cultured in Ham's F12 medium supplemented with 10% bovine serum (Sigma-Aldrich, St Louis, MO) and 0.25 mg/mL G418 sulfate (Invitrogen, Carlsbad, CA) in 5% CO_2_/95% air at 37°C.

### Membrane binding assay

Membranes were prepared from CHO-K1 cells stably expressing human S1P receptors based on the methods of Mandala *et al.* [35]. Briefly, cells were washed in PBS, suspended in 10 mM Tris-HCl (pH 7.5), 5 mM EDTA, and 1x Complete protease inhibitor cocktail (Roche Diagnostics, Mannheim, Germany), and were disrupted on ice using a polytron homogenizer. Following centrifugation at 80,000 × *g* for 40 min at 4°C, the pellet was suspended in 10 mM Tris-HCl (pH 7.5), 10% glycerol, and 1x Complete protease inhibitor cocktail and stored at −80°C. [^33^P]-S1P (American Radiolabeled Chemicals, St. Louis, MO) were used as radio-labeled ligands. Test compounds in assay buffer 1 (for S1P_1_, S1P_2_, S1P_3_, and S1P_5_; 50 mM Tris-HCl, pH 7.5, 5 mM MgCl_2_, 0.5% fatty acid-free BSA, and 1x Complete protease inhibitor cocktail) or assay buffer 2 (for S1P_4_ 50 mM Tris-HCl, pH 7.5, 100 mM NaCl, 15 mM NaF, 0.5% fatty acid-free BSA, and 1x Complete protease inhibitor cocktail) were added to a 96-well plate. Radio-labeled ligands and membranes were added to give a final volume of 200 µL. Binding was performed for 60 min at room temperature and was terminated by collecting the membranes onto unifilter GF/B plates (Perkin Elmer, Boston, MA) with a UniFilter96 Harvester (Perkin Elmer). After drying the filter plates for 30 min, filter-bound radionuclides were measured on a TopCount NXT microplate scintillation counter (Perkin Elmer). Specific binding was calculated by subtracting the radioactivity that remained in the presence of a 1000-fold excess of unlabeled S1P.

### Intracellular calcium measurement

CHO-K1 cells stably expressing hS1P_1_ or hS1P_3_ were plated at 2×10^4^ cells/well in 96-well plate and incubated for 2 days at 37°C in 5% CO_2_/95% air. Cells were loaded with Ham's F12 medium containing 5 μM Fura2-AM and 20 mM HEPES (pH 7.4) at 37°C for 1 h. After loading, cells were washed with Hanks solution containing 20 mM HEPES (pH 7.4), and stimulated with test compounds. Fluorescence intensity was measured by the ratio of emission fluorescence at 500 nm by excitation at 340 and 380 nm using a Fluorescence Drug Screening System (FDSS-6000, Hamamatsu Photonics K.K., Shizuoka, Japan).

### Mice

Six- to 7-week-old male Balb/c mice were purchased from Charles River Japan, Inc. (Kanagawa, Japan). Before use, mice were maintained in a specific pathogen-free animal facility Fat the Institute of Experimental Animal Sciences, Osaka University Graduate School of Medicine, and were kept in a room maintained at 25°C with a 12 h light/dark cycle and free access to food and water. All experiments were performed in accordance with the Guidelines for Animal Experiments of Osaka University. The protocol was approved by the Committee on the Ethics of Animal Experiments of Osaka University (approval ID: J003549-009). All surgery was performed under sevoflurane anesthesia, and all efforts were made to minimize suffering.

### Drug Treatment

Colitis was induced by 2.5% w/v DSS (MW: 5000, Wako, Osaka, Japan) in drinking water for 5 days. W-061 (Ono Pharmaceutical, Osaka, Japan) was dissolved in 0.5% w/v methylcellulose solution. The drug was administered orally at a dose of 3 mg/kg/day. After induction of colitis, daily clinical assessment of colitis was performed by measuring body weight and food intake, and observing fecal properties. Mice were sacrificed on day 3, 6 or 10.

### Isolation of lymphocytes

Lymphocytes were isolated from spleen (SP), mesenteric lymph nodes (MLN), Peyer's patches (PP) and lamina propria (LP) using a modification of the method reported by Atarashi *et al.* [36]. Briefly, single-cell suspensions were obtained by gently pressing the SP and MLN, filtered through 70-µm nylon meshes and suspended in HBSS (Wako) supplemented with 2% fetal bovine serum (FBS). Cells from lymphocytes were treated with RBC lysis buffer (eBioscience, San Diego, CA) before suspension. To prepare single-cell suspensions from PP, tissues were treated with RPMI 1640 (Sigma-Aldrich) containing 2% FBS and 1 mg/mL collagenase type II (Invitrogen) for 20 min at 37°C. For isolation from LP lymphocytes, intestines were opened longitudinally, washed with ice-cold PBS to remove fecal content, and were shaken in HBSS supplemented with 2% FBS and 5 mM EDTA for 20 min at 37°C. Intestines were then cut into small pieces and shaken with RPMI 1640 containing 2% FBS and 1 mg/mL collagenase type II for 25 min at 37°C. Digested tissues were resuspended with 8 mL of 40% Percoll (GE Healthcare, Waukesha, WI) and overlaid on 4 mL of 75% Percoll. Gradient separation was performed by centrifugation at 760 × g for 20 min at 37°C. Lymphocytes were collected at the interface of the Percoll gradient, washed and suspended in RPMI 1640 containing 2% FBS.

### Flow Cytometric Analysis

Anti-CD4 monoclonal antibody (mAb) (RM4-5), anti-IL-17A mAb (TC11-18H10.1), anti-IFN-γ mAb (XMG1.2) and anti-CD25 mAb (PC61) were purchased from Becton Dickinson, and anti-Foxp3 mAb (FJK-16s) was purchased from eBioscience.

Intracellular expression of IL-17, IFN-γ or Foxp3 in CD4^+^ T cells were analyzed using a Cytofix/Cytoperm Kit (Becton Dickinson) according to the manufacturer's instructions. Briefly, lymphocytes from SP, MLN, PP or LP were incubated with 50 ng/mL phorbol myristate acetate (Sigma), 5 µM calcium ionophore (Sigma) and Golgistop (Becton Dickinson) at 37°C for 4 h.

For Th17 and Th1 determination, surface staining for lymphocytes were performed with a cocktail of fluorescently labeled anti-CD4 mAb for 30 min at 4°C. Subsequently, cells were permeabilized with Cytofix/Cytoperm for 20 min at 4°C, and intracellular cytokine staining was performed with fluorescently labeled anti-cytokine antibodies for 30 min at 4°C. Similarly, for Treg determination, lymphocytes were stained with a cocktail of fluorescently labeled anti-CD4 mAb and anti-CD25 mAb. After Cytofix/Cytoperm treatment, cells were stained with fluorescently labeled anti-Foxp3 mAb. Stained lymphocytes were analyzed on a FACSCalibur Flow Cytometer (Becton Dickinson) using Cell Quest software. Among lymphocytes, CD4^+^ IL-17^+^ cells were considered to be Th17. Similarly, CD4^+^ IFN-γ^+^ lymphocytes were considered to be Th1, and CD4^+^ CD25^+^ Foxp3^+^ lymphocytes were considered to be Treg.

### Histological Analysis of Colon

Histological analysis was performed on colon divided into three areas; proximal, medial and distal. Tissue was fixed in 4% paraformaldehyde in PBS and embedded in paraffin. Sections (2 µm) were prepared and stained with hematoxylin and eosin. Histopathologic changes in the colonic mucosa were semi-quantified according to a modified scoring system ^18^: (a) cellular infiltration in the lamina propria (scored from 0 to 3); (b) mucin depletion (scored from 0 to 2); (c) crypt abcesses (scored from 0 to 2); (d) epithelial erosion (scored from 0 to 2); (e) hyperemia (scored from 0 to 3); and (f) thickness of mucosa (scored from 1 to 3).

### Statistical analysis

All data are expressed as means ± SD. Data were analyzed by two-tailed Student's *t* test. Values of *p*<0.05 were considered to be significant.

## Supporting Information

Figure S1 **The change in body weight was not affected with daily administration of W-061 (3 mg/kg).** Data represent absolute values (means ± SD, n = 6).
(TIF)
