# Supplementary figures and images for: Therapeutic Effects of Novel Sphingosine-1-Phosphate Receptor Agonist W-061 in Murine DSS Colitis
Source: PLoS One. 2011 Sep 8;6(9):e23933. doi: 10.1371/journal.pone.0023933 (PMC3169557; doi:10.1371/journal.pone.0023933)

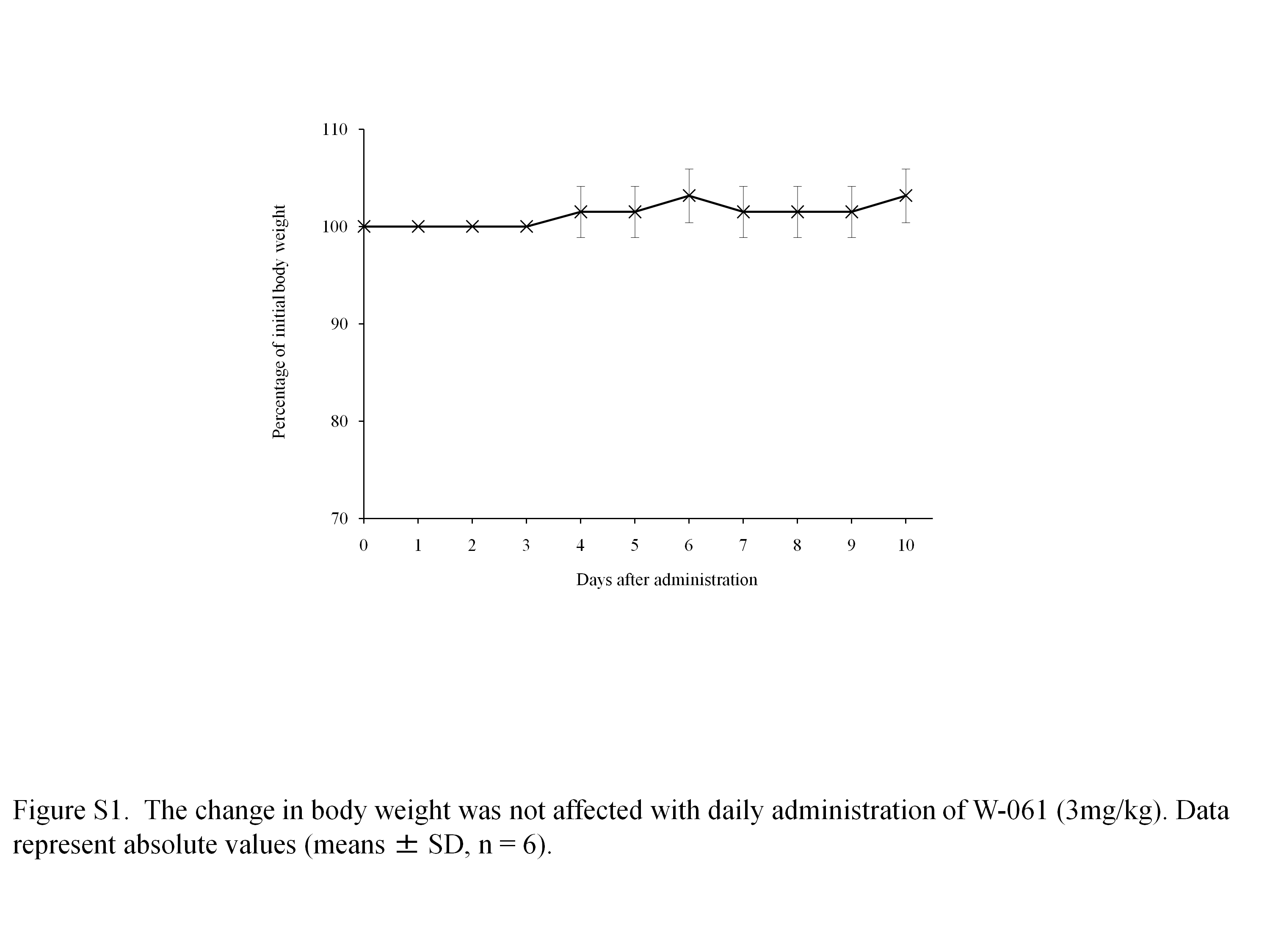

Supplement: Figure S1 — The change in body weight was not affected with daily administration of W-061 (3 mg/kg). Data represent absolute values (means ± SD, n = 6). (TIF) [file pone.0023933.s001.tif]
